# COVID-19, social identity, and socially responsible food consumption between generations

**DOI:** 10.3389/fpsyg.2023.1080097

**Published:** 2023-03-08

**Authors:** Sandra Nelly Leyva-Hernández, Antonia Terán-Bustamante, Antonieta Martínez-Velasco

**Affiliations:** ^1^Facultad de Ingeniería y Negocios San Quintín, Universidad Autónoma de Baja California, San Quintín, Mexico; ^2^Facultad de Ciencias Económicas y Empresariales, Universidad Panamericana, Mexico City, Mexico; ^3^Facultad de Ingeniería, Universidad Panamericana, Mexico City, Mexico

**Keywords:** stimulus-organism-response, health belief model, generation Z, generation Y, generation X, sustainability, environment

## Abstract

**Introduction:**

The objective of the research was to analyze the effect of COVID-19 with the predictors of the health belief model (perceived severity, perceived benefits, and cue to action) on the social identity of the consumer and the social identity of the socially responsible food consumption among four generation groups of adults based on the stimulus-organism-response model.

**Methods:**

The study had a quantitative approach explanatory design and a cross-sectional temporal dimension. A total of 834 questionnaires were collected from adults in the metropolitan area of Mexico City, and the data were analyzed through partial least squares structural equation modeling.

**Results:**

The results indicated that perceived severity, perceived benefits, and cue to action positively and significantly influenced social identity, and this positively and significantly influenced socially responsible consumption. In addition, identity was found to be a variable that had a total mediation effect between perceived severity and socially responsible consumption, perceived benefits and socially responsible consumption, and cue to action and socially responsible consumption. While the perceived barriers only had a direct effect on socially responsible consumption. Likewise, a difference was found between generation X and Y, generation Z and X, and generation Y and X in the relationship between cue to action, belonging to a social network group, and social identity.

**Discussion:**

In this sense, these results allow us to consider that when environmental stimuli (predictors of the health belief model) affect the organism (social identity), it will respond with socially responsible food consumption. This type of consumption is explained through social identity and is modified according to the age of the consumers due to the effects of social networks.

## 1. Introduction

In recent decades, sustainability initiatives and strategies have been launched focused on combating climate change. In this sense, the circular economy (CE) aims to contribute to the current ecological transition, providing economic advantages and preserving the global society for future generations; among these initiatives is socially responsible food consumption. The agri-food industry generates significant carbon emissions that cause environmental damage and depletion of natural resources (Abbate et al., 2023). Therefore, many experts believe that the existing food and agricultural system is unsustainable (Campbell et al., 2017; Abbate et al., 2023), for which the redesign of value creation in businesses is necessary firms to reduce the use of resources and generation of pollutants (Abbate et al., 2023). Added to the above is food waste, derived its consumption or non-consumption (Rasool et al., 2021). Therefore, global food security is a critical concern for the entire world (Lombardi et al., 2019), and a primary area of the circular economy (CE; Fassio and Tecco, 2019).

According to Prothero et al. (2011), Sun et al. (2021), and Balaji et al. (2022), consumers are critical to the transition to socially responsible sustainable consumption. Therefore, analyzing people’s behavior, especially of the new generations, regarding food consumption is relevant, to generate actions focused on this objective.

Currently, socially responsible consumption is identified as part of the trajectory for sustainable development. In other words, the forms of production, distribution, and consumption of food cannot ignore sustainability, as well as the perception related to the consumer (Peano et al., 2019).

When talking about responsible eating, we must refer to a healthy diet, ideal for preventing diseases and respecting the environment. Conversely, poor nutrition can reduce immunity, increase vulnerability to disease, impair physical and mental development, and reduce productivity (FAO et al., 2021).

Derived from the COVID-19 pandemic, the population begins to worry more about their health and prefer foods that benefit the consumer, the producer, and the environment (Brugarolas et al., 2020). That represents an advantage at this time for the socially responsible consumption of food.

In turn, young adults are critical to this type of analysis since they mainly demand better environmental quality (Nieves, 2016). In addition, millennial young adults want to improve the environment and seek to consume sustainable products (Peñalosa and López, 2016). Likewise, young undergraduate and graduate consumers around 24 years of age are a crucial segment in the consumption of these products (Pham et al., 2019).

In Mexico, local markets represent alternatives for the commercialization of socially responsible products (Roldán et al., 2016). Therefore, marketing networks emerge following this orientation, such as the Mexican Network of Tianguis and Organic Markets, which promotes fair trade in food between producers and consumers (Bustamante-Lara and Schwentesius-Rindermann, 2018).

However, due to the health contingency, there are restrictions on physical marketing due to social distancing and the closure of physical stores (Sheth, 2020). For this reason, electronic commerce has significantly increased (Cavallo et al., 2020), since people keep their purchases without compromising their health. In addition, one of the challenges to sustainable food consumption is promoting places of sale (Oliveira et al., 2021). Therefore, social networks offer a convenient alternative for both the promotion and sale of sustainable products and thus positively affect the socially responsible food consumption.

In addition, COVID-19 has changed the motivations for purchasing behavior. For example, during this health contingency, the variables influencing the intention of socially responsible food consumption are mainly attitude (Cachero-Martínez, 2020). Furthermore, organic purchase intention is also explained by personal attitudes, perceived social pressure, and perceived consumer autonomy during the pandemic (Latip et al., 2020). However, this disease’s impacts on socially responsible consumption or social networks have not been evaluated.

Nor is much known about the types of socially responsible consumers that emerged due to this pandemic or about the differences in generational consumption of young adults. Therefore, the objective of the research is to analyze the effect of COVID-19 with the predictors of the health belief model (perceived severity, perceived benefits, and cue to action) on the social identity of the consumer and the latter on the socially responsible consumption of foods among four generational groups of adults based on the stimulus-organism-response model.

The acquisition of healthy and safe products is a fundamental right that consumers have, and public institutions and companies are responsible for ensuring this right is fulfilled. However, the consumer must also be concerned about compliance with this principle and ensure that the products purchased are healthy and safe for himself/herself, all those involved in product manufacturing process, and our planet, in particular.

According to the above, this research aims to analyze the effect of COVID-19 on the diet and health of four generations of adults. Furthermore, the predictors of the model of health beliefs (perceived severity, perceived benefits, and key to action) on the consumer’s social identity and socially responsible food consumption are analyzed.

## 2. Theoretical framework

### 2.1. Stimulus-organism-response model

Some models extend the understanding of sustainable consumption, such as the stimulus-organism-response model, which examines cognitive and affective influences on behavior as external stimuli that affect the internal state and, consequently, result in behavior (Mehrabian and Russell, 1974). Similarly to theory of planned behavior of Ajzen (1991), the stimulus-organism-response model seeks to explain an individual’s behavior. However, unlike said theory, in the stimulus-organism-response model, factors external to the individual are the predictors of the individual’s internal state, which is a predictor of behavior. While in the theory of planned behavior attitudes, subjective norms and the perceived control of behavior are the predictors of individual’s behavior.

In food consumption, external stimuli that affect the internal state of the consumer and consequently lead to food purchasing behavior have been considered in various ways (Lee and Yun, 2015; Liu and Zheng, 2019; Lee et al., 2020). For example, it is analyzed how objects and psychological stimuli affect the individual’s internal state, and the individual as a response has a food sustainable consumption (Lee and Yun, 2015). Liu and Zheng (2019) analyze how stimuli (food safety incidents, consumer environment orientation, and consumer health orientation) influence consumer cognition, influencing organic purchasing. Through the stimulus-organism-response model, Lee et al. (2020) explain the purchasing behavior of organic food through the stimulation of the intrinsic and extrinsic characteristics of the food in the consumer’s attitude and the effect of this on shopping behavior.

Manthiou et al. (2017) consider that the physical environment (stimulus) influences the cognitive and emotional perspectives of the consumer (organism) responding with the behavior towards the environment (response). Therefore, the COVID-19 pandemic can be considered the physical environment, the stimulus. In this sense, the stimulus-organism-response model is used as a theoretical framework to analyze the purchasing behavior of organic food during the health contingency period due to COVID-19 (Liu et al., 2021; Yin et al., 2021). However, not all studies analyze the possible impacts of this disease on consumption; they only analyze consumption in the context of the pandemic without quantifying its effect (Liu et al., 2021). Yin et al. (2021) consider COVID-19 as the external stimulus through the event force that the pandemic has on organic food consumption. However, unlike this, in this research, the external stimuli of COVID-19 are analyzed through the health belief model. This model is used in research related to healthcare behaviors (Wong et al., 2020; Guidry et al., 2021; Mercadante and Law, 2021).

### 2.2. External stimuli from the health belief model, and social identity (organism)

The health belief model (HBM) explains preventive health behaviors through personal motivation to achieve goals in the area of health (Maiman and Becker, 1974). In addition, I HBM aims to analyze behaviors in conditions of uncertainty (Becker et al., 1974), such as what is happening with the COVID-19 pandemic.

The Health Belief Model postulates that for the individual to have behavior related to preventive health, they must have the disposition to act based on the perception of vulnerability to a health condition and the severity of the consequences of contracting the condition. Furthermore, assessment of the feasibility and efficacy of reducing their exposure by performing the behavior is better than the barriers and costs. A cue to action is triggered as their interpersonal interactions (Maiman and Becker, 1974).

Likewise, Rosenstock et al. (1988) argue that individuals must have sufficient motivation that the health condition is relevant and that they are susceptible to it. Moreover, a health recommendation will benefit them since it will reduce their susceptibility to an acceptable cost, the barriers, which are not necessarily only economic.

Since the HBM, some authors consider perceived severity, benefits, and perceived barriers predictors of health-related behavior (Myers and Goodwin, 2011; Guidry et al., 2021). These factors related to the characteristics and knowledge of the individual affect their beliefs and encourage behavior (Mercadante and Law, 2021). In addition, studies prove that external stimuli, such as perceived severity, affect the organism under the analysis of the SOR model (Wang et al., 2021).

It is also necessary to contemplate the signals for action (cue to action) included within the health belief model as stimuli of the organism, which can be interpersonal interactions or with the media that provide individuals with knowledge about the health condition (Maiman and Becker, 1974). These may be social networks because they provide information to those who interact with them without topics such as recommendations and are predictors of consumer behavior (De Valck et al., 2009). Zaglia (2013) confirms that social interactions between members of a social network and belonging to that network influence consumers’ social identity. Therefore, according to the stimulus-organism-response model, it is possible to consider the predictors of the health belief model as the external stimuli caused by COVID-19 that affect the organism (social identity). With this, the following hypotheses are postulated:

*H1a*: Perceived severity positively and significantly influences social identity.

*H1b*: Perceived benefits positively and significantly influence social identity.

*H1c*: Perceived barriers positively and significantly influence social identity.

*H1d*: The cue action (social networks) positively and significantly influences social identity.

### 2.3. Social identity (organism) and socially responsible consumption (response)

Identity is defined as a consumer association towards a label they choose and a clear image of how the person looks, thinks, and feels (Reed et al., 2012). Finally, in green consumption, pro-environmental self-identity is defined as the consumer morally obliged to carry out a green action that will bring satisfaction (Mutum et al., 2021).

Mutum et al. (2021) find that identity explains green shopping; if the consumer considers himself concerned and respectful of the environment, it causes him pride and pleasure to be considered a compliant consregarded as consumer will make green purchases regularly. In addition, according to Liu et al. (2021), the SOR model allows for analyzing consumer behavior as it provides a structured framework to evaluate the environmental stimulus in the consumer’s psychological factors such as emotion, perception, and cognition and turn their effect on consumption. Therefore, in this research, the following hypothesis is proposed:

*H2*: Social identity positively and significantly influences socially responsible consumption.

### 2.4. External stimuli and socially responsible consumption (response)

An individual’s perceived risk is a prospective subjective loss that could endanger their health and well-being (Paek and Hove, 2017; Chen and Wang, 2022). In a crisis, consumers respond to risk based on their subjective perception since their knowledge of risk factors lacks objectivity (Paek and Hove, 2017; Lejano and Stokols, 2021). According to Slovic et al. (1984) when there is an unknown risk, people perceive that the dangers are newcomer, and unobservable, similar to what happens in the context of the pandemic. Since the pandemic, some research find that perceived risk affects the intention to purchase food, whether online or in person (Leung and Cai, 2021; Chen and Wang, 2022).

While a person’s perception of the seriousness of a threat and how it will affect them is known as perceived severity (Milne et al., 2000; Baghiani-Moghadam et al., 2015). Therefore, the perceived severity denotes how much the perceived risk, in this study, COVID-19, can affect the person. In research in the area of health, the perceived severity affects the decisions to carry out behavior that brings benefits to health, as the health belief model proposes since the perceived risk affects the intention to vaccinate (Myers and Goodwin, 2011; Guidry et al., 2021). During the health contingency by COVID-19, perceived severity positively influences the intention to purchase organic food. The negative impact of the disease leads consumers to be willing to buy organic food when shopping (Wang et al., 2021). For that, the following hypothesis is proposed:

*H3a*: Perceived severity positively and significantly influences socially responsible consumption.

Socially responsible consumption guides actions toward improving people’s quality of life and caring for the environment. Therefore, for sustainable food consumption to exist, it is required that it be economically and ecologically viable, that is, that food is accessible to the consumer, has a fair price, and does not deteriorate the environment. A relevant variable is the perception of personal gain, that is, the perceived benefits. This is conceptualized as the people’s perception advantages and disadvantages of being socially responsible (Ellen et al., 1991; Ellen, 1994). The perception of personal benefit refers to the subjective assessment that the individual makes about the personal advantages and disadvantages unique has when acting in asocially responsibly (Lin and Hsu, 2015).

In this sense, the behavior of the socially responsible consumer is explained by certain beliefs of perceived personal benefit (Zhao et al., 2014; Lin and Hsu, 2015; Lin and Niu, 2018; Pawaskar et al., 2018; Testa et al., 2019; Yarimoglu and Binboga, 2019). When there are health benefits for certain foods or beverages, such as coffee, consumers are more inclined towards their consumption; this phenomenon occurs when it comes to female consumers (Samoggia and Riedel, 2019). Therefore, the following hypothesis is proposed:

*H3b*: Perceived benefits positively and significantly influence socially responsible consumption.

Promote a healthier lifestyle, there are both benefits and barriers. The barriers consumers perceive can be economic when there are significant differences in food prices (The European Food Information Council, 2009). The barriers can also be personal when they attend to the lack of time both to travel to those places where to find this type of sustainable products and to prepare them, which has brought with it a restructuring of eating habits, due to the growing consumption fast food (NESI Forum on New Economy and Innovation, OCU, 2019). There are also systematic barriers that refer to the lack of reliable information on products, the lack of confidence in company social responsibility policies, the planned obsolescence of products that force them to be replaced by others, and due to the lack of legislation that acts as a boost to responsible consumption (Lima et al., 2021). Finally, it can find the barriers of eating habits that refer to resistance to change, since the patterns of adults have been formed for a long time and are difficult to change (Munárriz and De Luis, 2009; Leng et al., 2017). When there are health benefits to performing a behavior such as reducing meat consumption, the perceived benefits and barriers influence the intention to perform such behavior (Cheah et al., 2020).

*H3c*: Perceived barriers positively and significantly influence socially responsible consumption.

Cue to action involves personal interactions and participation in social groups (Maiman and Becker, 1974; De Valck et al., 2009). Being a member of different groups in social networks causes consumers to acquire sustainable purchasing behavior. For example, they choose products with green packaging buy green products or verify the products’ ingredients to ensure that their purchase is sustainable (Cui et al., 2022). The above propose the following hypothesis:

*H3d*: The cue action (social networks) positively and significantly influences socially responsible consumption.

### 2.5. The mediation effect of the social identity

Generally, identity is a predictor of sustainable consumption behaviors (Mutum et al., 2021), but it can act as mediator on this behavior. In socially responsible consumption, identity is considered a predictor of socially responsible purchasing behavior in young adults (Johnson and Chattaraman, 2021). According to the stimulus organism response model, the stimulus leads the organism to have a response that can be a behavior (Mehrabian and Russell, 1974). From an extension of the SOR, Talwar et al. (2021) find that the consumer’s identity as an ethical person predicts their willingness to purchase organic food. However, this study also seeks to know if the stimulus affects behavior through the organism, as evidenced by Liu et al. (2021). They find that the organism (cognition) acts as a mediators in the relation of stimulus and organic food purchasing behavior. Additionally, Wang et al. (2021) find that the organism (health consciousness) has a mediating effect between the stimulus (perceived severity) and the response (purchase intention to organic food). Therefore, social identity can take the role of mediator between perceived severity and socially responsible consumption and with this the following hypothesis is postulated:

*H4a*: Social identity significantly mediates the relationship between perceived severity and socially responsible consumption.

To achieve a healthy and sustainable lifestyle, the consumer considers the benefits and barriers involved in their purchase as stated above, an assessment of the advantages and disadvantages of having a socially responsible behavior is made (Ellen et al., 1991), however, little is known about what affects or intervenes in these relationships. The barriers to socially responsible behavior can be economic, personal, or habitual (Munárriz and De Luis, 2009; The European Food Information Council, 2009; Leng et al., 2017), while the benefits are generally towards health (Samoggia and Riedel, 2019). In addition, through the SOR it is possible to consider mediation of the organism between the stimulus and the response (Liu et al., 2021; Wang et al., 2021).

For this reason, this research explores whether social identity has a role as a mediator between benefits and socially responsible consumption and barriers and socially responsible consumption, given that identity also explains ecological consumption and the SOR model provided the theoretical framework for their analysis (Liu et al., 2021; Mutum et al., 2021). Therefore, the following hypotheses are proposed. Identity mediates the relationships between stimuli (perceived benefits, perceived barriers) and socially responsible behavior.

*H4b*: Social identity significantly mediates the relationship between perceived benefits and socially responsible consumption.

*H4c*: Social identity significantly mediates the relationship between perceived barriers and socially responsible consumption.

Also, according to the health belief model, there must be a cue to action so that the individual can have a behavior that is good for him when there is a condition of risk to his/her health (Maiman and Becker, 1974), as in the case of COVID-19. Although, like the previous cases, little is known about the interactions that can affect this relationship, research explores whether social identity can mediate this relationship since this is also a variable that explains similar behavior (Mutum et al., 2021). Therefore, the following hypothesis is postulated:

*H4d*: Social identity significantly mediates the relationship between cue action (social networks) and socially responsible consumption.

### 2.6. Generational change

Age is a variable that act as a predictor or moderator variable in the analysis of sustainable consumption (Chekima et al., 2016; Bulut et al., 2017). Bulut et al. (2017) find age to be a predictor of sustainable consumption in Turkey, while Chekima et al. (2016) find that age can act as a moderating variable of sustainable consumption in Malaysia. In addition, Quoquab and Mohammad (2020) in a sustainable consumption review from 2000 to 2020, propose age as a moderating variable in a conceptual model. In addition, age also acts as a moderating variable when analyzing the effect of COVID-19 on both sustainable consumption and social responsibility, as shown in the study by Ali et al. (2021). Their study confirms significant differences between generations X, Y, and baby boomers in the relationships between COVID-19 and sustainable consumption and COVID-19 and the social responsibility of Malaysian consumers. Therefore, when studying the effect of COVID-19 on socially responsible consumption, age can have a moderating effect on the relationships in the model, as proposed in the following hypothesis:

*H5*: There is a categorical moderation effect of the generational group in the relationships between the model’s constructs.

**Figure 1:**
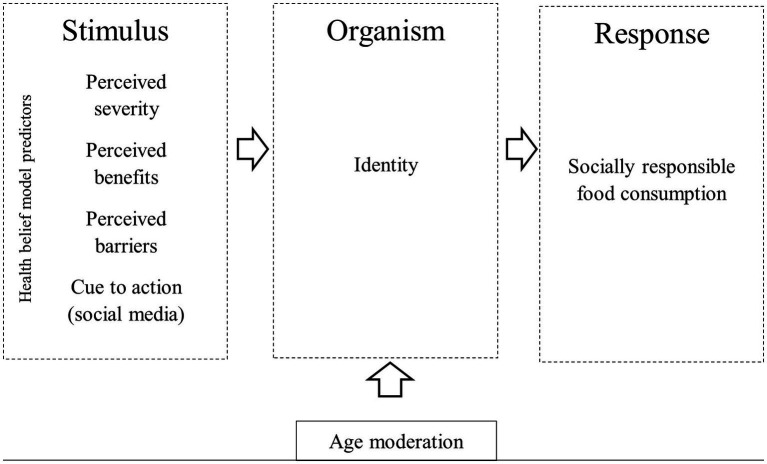
Research conceptual model.

In this way, the stimulus-organism-response model and the health belief model allow us to analyze the effect of COVID-19 on socially responsible consumption, as shown in Figure 1.

## 3. Methodology

The study had an exploratory approach since the factors that affect socially responsible consumption were analyzed from two psychological theories: health belief model and SOR. The temporal dimension of the study was cross-sectional. A sample of 834 adults from the metropolitan area of Mexico City was collected from August 19 to September 12, 2022. This research followed an approach to avoid the disproportionate representation of socially responsible consumers. As Yadav and Pathak (2016) recommended, the study does not include selection criteria for random sampling that will segment a specific sector or type of consumption. Therefore, only people over 18 who resided within the metropolitan area of Mexico City were chosen. To verify the accuracy of the model, an analysis of an alternative conceptual model was newcomer carried out. In addition, before this, three academic experts in the area were interviewed to verify the chosen model.

The treatment of the data was carried out through Partial Least Squares Structural Equation Modeling (PLS-SEM). The type of analysis that was carried out included mediation analysis performed with a Bootstrapping analysis to calculate the significance of the effects, multigroup analysis (MGA) with a Bootstrapping analysis to determine the path coefficients of the groups, and determining the differences of the groups (Henseler et al., 2009), and calculation of the measurement invariance of composite models (MICOM) for guarantee the validity of the MGA that can be carried out by PLS-SEM (Hair et al., 2017, 2021). Unlike other methodologies, such as the choice experiment used in consumer analysis with responsibility initiatives (Boccia and Sarnacchiaro, 2020), the structural equation modeling allows the evaluation of latent variables that are measured through other variables, called manifest variables (Hair et al., 2017), which makes it possible the use of structured questionnaires in data collection. In addition, PLS-SEM allows mediation and multigroup analysis (Hair et al., 2021). SmartPLS version 4 software was used for data analysis (Ringle et al., 2022).

The sample included four generations from Z to baby boomers as shown in Table 1. Generation z had the most remarkable presence in the sample with 61.63%. Participation between women and men was almost balanced, with women with 48.08% participation and men with 42.33%. Most participants had undergraduate studies (69.06%) and were students (55.76%).

**Table 1:** Sociodemographic data.

Variable		Frequency	Percentage
Age	Generation Z	514	61.63%
	Generation Y	122	14.63%
	Generation X	168	20.14%
	Baby boomers	28	3.36%
Gender	Female	401	48.08%
	Male	353	42.33%
Scholarship	Secondary	15	1.80%
	High school	171	20.50%
	Bachelor’s degree	576	69.06%
	Master’s degree	52	6.24%
	Doctorate	20	2.40%
Occupation	Student	465	55.76%
	Employee	213	25.54%
	Entrepreneur	61	7.31%
	Businessman	51	6.12%
	None	35	4.20%
	Retired	9	1.08%

### 3.1. Study measures

Perceived severity has been defined as the perception of the seriousness of the consequences of contracting a condition that is detrimental to health (Maiman and Becker, 1974). For this study, this concept was adapted according to the research objective, so perceived severity was defined as the consumer’s perception of the seriousness of the consequences of contracting the COVID-19 disease. For its measurement, the items proposed by Myers and Goodwin (2011) were adapted to four items with a Likert scale from 1 totally disagree to 5 totally agree.

Perceived benefits were defined as the perceived feasibility and efficacy of reducing the consumer’s vulnerability to contracting the COVID-19 disease by engaging in socially responsible food purchasing behavior (Maiman and Becker, 1974). The variable was adapted from the scale used by Myers and Goodwin (2011) measured by three items from 1 totally disagree to 5 totally agree.

The perceived barriers were defined as the limitations to carrying out socially responsible food purchase behavior due to time and ignorance. For their measurement, the items proposed by Nguyen et al. (2016) were adapted to 2 items with a Likert scale of 5 points (1 totally disagree to 5 totally agree). Initially, 3 items were considered to measure this variable, however, since one of them during the pilot test had a factor loading of less than 0.7, it was discarded from the model. This item measured the financial barrier. Some authors have validated using 2 items measuring variables (Baumert et al., 2014; Forsell et al., 2019). Also, it is recommended improve scale items to remove ambiguity as procedural remedies to prevent common method bias (Podsakoff et al., 2012). Therefore, to measure this variable only 2 items were used.

The cue to action was defined as the interpersonal interactions that consumers have within groups where they obtain behavioral information (Maiman and Becker, 1974), which, in the case of this study, these groups are social networks. This construct was adapted from Cui et al. (2022) with an item with a Likert scale from 1 totally disagree to 5 totally agree.

Social identity was conceptualized as the set of attributes perceived by the individual that represents their way of thinking, feeling, and being (Stets and Biga, 2003). The Johnson and Chattaraman (2021) scale was adapted to 4 items measured at 5 points (1 totally disagree to 5 totally agree).

The measurement of the socially responsible food consumption construct was adapted and conceptualized by Villa Castaño et al. (2016) as the recognition of the consumer that the company is responsible for the effects caused by the production of its food towards the environment or vulnerable groups. This construct was measured by three items with a Likert scale from 1 totally disagree to 5 totally agree. Table 2 shows the constructs of the research model with their measurements.

**Table 2:** Measurements.

Construct	Item		Author
Identity	IDEN 1	Being socially responsible is an important part of who I am	Johnson and Chattaraman (2021)
	IDEN 2	Social responsibility is something about which I have a clear feeling.	
	IDEN 4	I think about social responsibility.	
	IDEN 5	Socially responsible food consumption is essential to me as an individual.	
Perceived barriers	INCPER2	While shopping, I need help to easily distinguish between conventional (heavily processed) and fair trade or organic or agroecological foods.	Nguyen et al. (2016)
	INCPER3	I need much extra time to buy agroecological food.	
Perceived benefits	BENPER1	My organic-based diet reduces my worries about contracting COVID-19.	Myers and Goodwin (2011)
	BENPER2	The consumption of fair-trade food reduces the possibility of contracting COVID-19 or its complications.	
	BENPER3	If I eat agroecological food, I will reduce the probability of being hospitalized for COVID-19.	
Perceived severity	SEVPER2	I will be very fragile if I contract COVID-19.	Myers and Goodwin (2011)
	SEVPER4	COVID-19 altered my health.	
	SEVPER5	COVID-19 altered my eating habits.	
Socially responsible consumption	CSREXT3	I make an effort to support and buy from food companies that practice waste management and recycling.	Villa Castaño et al. (2016)
	CSREXT4	I try to support and buy from food companies that promote clean production and avoid polluting the environment.	
	CSREXT7	I try to support and buy from food companies that promote local or agroecological food to support local businesses.	
Cue to action	SOCMED5	I am a member of different groups in social networks where people sell or consume organic food.	Cui et al. (2022)

### 3.2. Data analysis

The sample size (834) met the minimum required for the PLS-SEM analysis, which was obtained by a statistical power analysis using Cohen’s statistical power tables suggested by Benitez et al. (2020) when using PLS-SEM. That consisted of determining (1) the level of significance of the acceptable study, which was 0.05; (2) the number of predictors, considered as the most significant number of structural paths of the endogenous construct, which was 5; and (3) the effect size, which was small to have a conservative approach to the study (Cohen, 1988; Nitzl, 2016; Benitez et al., 2020). With these values, according to the statistical power tables, the minimum size required was 647 (Nitzl, 2016), the study sample size being greater than that calculated.

Data analysis was performed using PLS-SEM because it is recommended to use this method of analysis when a theoretical framework is tested, and there is a complex structural model with several constructs and indicators (Hair et al., 2019). Before analyzing the model with the total sample, a pilot test was carried out to validate the measurement scales and determine their reliability, convergent, and discriminant validity, for which items with low factor loads were eliminated, after which the data was collected total sample number. Before data analysis, it was confirmed that the data did not have an excessively abnormal distribution with kurtosis and asymmetry values outside the range of −1 to 1 (Hair et al., 2017). Therefore, first, the assessment of the measurement model was carried out, and second, the assessment of the structural model was carried out as postulated by Hair et al. (2019). The fit of the model was also evaluated (Benitez et al., 2020), and advanced analyzes were performed to complete the hypothesis tests involving mediation analysis and multigroup analysis to assess the moderating effect of age on the model of research (Nitzl et al., 2016; Wong, 2016). Analyzes made in this research are shown in Table 3.

**Table 3:** The research analyzes through PLS-SEM.

Analyses		Author
Assessment of the reflective measurement model	*Reliability of the indicators Internal consistency: *Composite reliability (rho_c), Cronbach’s alpha, and Dijkstra and Henseler’s value (rho_a) *Convergent validity: average variance extracted *Discriminant validity: Heterotrait-Monotrait Ratio	Dijkstra and Henseler (2015), Hair et al. (2017, 2019), Ali et al. (2018), Benitez et al. (2020)
Assessment of the structural model	*Determination coefficients (*R*^2^) *Effect sizes (*f*^2^) *Path coefficients	Benitez et al. (2020)
Mediation analysis	*Direct effects *Indirect effects	Zhao et al. (2010), Nitzl et al. (2016)
The fit of the model	*Standardized mean square residual	Benitez et al. (2020)
Multigroup analysis	*Measurement invariance of composite models *Multigroup analysis (MGA)	Henseler et al. (2009, 2016), Hair et al. (2021)

### 3.3. Common method bias

When more than one measurement of the same or different traits is taken using the same method, it is known as common method bias (CMB). It is thought to cause the discrepancy between the trait and measured scores (Podsakoff et al., 2012). Therefore, given that bias may alter outcomes due to systematic errors, CMB could signify a hazard in social scientific research (Schwarz et al., 2017). When the same method is used to measure different constructs, they could share some of the observed covariation (Podsakoff et al., 2012). Therefore, it is recommended to use procedural remedies to prevent CMB during the research design phase when analyzes the data by PLS-SEM like improving scale items to eliminate ambiguity (Podsakoff et al., 2012; Felipe et al., 2017).

A full collinearity test using variance inflation factors (VIF) was conducted to identify a potential CMB scenario (Kock, 2015; Felipe et al., 2017). Collinearity occurs when two or more variables measure the same attribute and is measured in models with multiple variables to avoid redundancy (Kock and Lynn, 2012). Calculating the scores of the latent variables in PLS-SEM does not eliminate the collinearity between them, although they have passed validity and reliability tests, it only minimizes the collinearity (Chin et al., 2003; Haenlein and Kaplan, 2004; Kock and Lynn, 2012). Vertical and lateral collinearity were assessed (Kock and Lynn, 2012; Felipe et al., 2017). Vertical collinearity was evaluated among latent variable predictors (Kock and Lynn, 2012), the results are shown in Table 4. To assess the lateral collinearity a *dummy* variable obtained with random values was used as an endogenous variable and the other variables of the model were the exogenous variables as recommended by Kock and Lynn (2012) (Table 5). All VIF values were less than 3.3, which according to Kock (2015) and Felipe et al. (2017) indicates that there are no multicollinearity problems and no CMB.

**Table 4:** Vertical collinearity test.

	Social identity	Socially responsible consumption
Cue to action	1.086	1.107
Social identity		1.105
Perceived barriers	1.077	1.080
Perceived benefits	1.123	1.152
Perceived severity	1.121	1.136

**Table 5:** Lateral collinearity test.

Cue to action	Social identity	Perceived barriers	Perceived benefits	Perceived severity	Socially responsible consumption
1.113	1.252	1.053	1.090	1.036	1.246

## 4. Results

### 4.1. Measurement model assessment

The evaluation of the reflective measurement model was carried out, which involves the assessment of the reliability of the indicators and the reliability of internal consistency, and the convergent and discriminant validity of the measures of the constructs (Ali et al., 2018). The indicators were considered to have values greater than 0.7 so that each would explain at least 50% of the variance of their construct (Benitez et al., 2020). Composite reliability, Cronbach’s alpha, and Dijkstra and Henseler’s value were used to assess internal consistency (Dijkstra and Henseler, 2015; Ali et al., 2018; Hair et al., 2019). It was taken into account that the internal consistency values were between 0.6 and 0.95 since values greater than 0.6 are considered acceptable in exploratory research and values greater than 0.95 suggest multicollinearity (Hair et al., 2019). To evaluate the convergent validity values greater than 0.5 of the average variance extracted (AVE) were taken as valid since this indicates that at least the set of indicators of the construct explains it by 50% (Hair et al., 2017; Ali et al., 2018). Hair et al. (2019) indicate that the Heterotrait-Monotrait Ratio (HTMT) is used to evaluate and guarantee the constructs’ discriminant validity. The values must be less than 0.85 when the constructs are conceptually different, as in the case of the constructs of this study. The results of the evaluation of the measurement model are shown in Table 6.

**Table 6:** Measurement model evaluation results.

Construct	Item (Load)	Cronbach’s alpha	Composite reliability (rho_a)	Composite reliability (rho_c)	The average variance extracted (AVE)	Heterotrait-Monotrait Ratio				
						Cue to action	Identity	Perceived barriers	Perceived benefits	Perceived severity
Identity	IDEN 1 (0.888)	0.910	0.911	0.937	0.787	0.212				
	IDEN 2 (0.914)									
	IDEN 4 (0.894)									
	IDEN 5 (0.852)									
Perceived barriers	INCPER2 (0.842)	0.699	0.729	0.867	0.766	0.157	0.153			
	INCPER3 (0.907)									
Perceived benefits	BENPER1(0.890)	0.862	0.879	0.915	0.782	0.247	0.258	0.234		
	BENPER2(0.885)									
	BENPER3(0.878)									
Perceived severity	SEVPER2 (0.749)	0.723	0.732	0.844	0.644	0.234	0.240	0.313	0.317	
	SEVPER4(0.854)									
	SEVPER5(0.802)									
Socially responsible consumption	CSREXT3(0.923)	0.892	0.897	0.933	0.823	0.202	0.668	0.217	0.221	0.215
	CSREXT4(0.933)									
	CSREXT7(0.864)									

### 4.2. Structural model assessment

**Figure 2:**
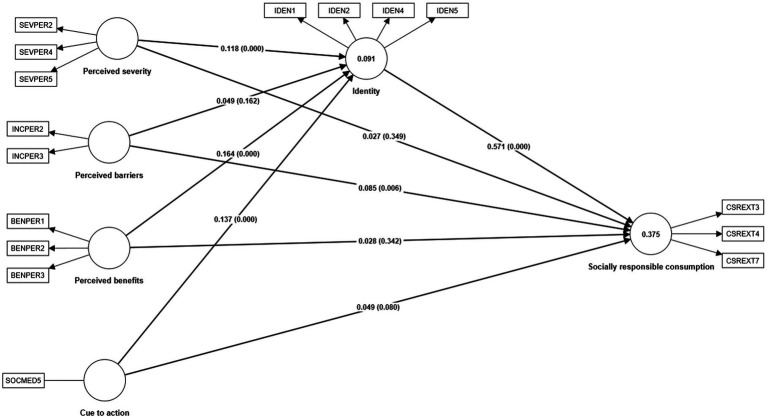
Structural model.

In the structural model assessment, the determination coefficients (*R*^2^), the effect sizes (*f*^2^), and the path coefficients were determined (Benitez et al., 2020), with which the tests of the hypotheses 1a, 1b, 1c, 1d 2, 3a, 3b, 3c, and 3d were carried out. Figure 2 shows the values of R^2^; the value of socially responsible consumption was weak because it had a value of less than 0.5 and greater than 0.25 (Hair et al., 2011). However, the effect size of social identity in socially responsible consumption is large (*f*^2^ = 0.474) because it is greater than 0.35 (Cohen, 1988) as shown in Table 7. Only perceived benefits have a small effect on social identity (*f*^2^ = 0.27). According to Cohen (1988) values greater than 0.25 of effect size are considered small, while the other values do not represent any effect.

**Table 7:** Results of the structural model assessment.

Hypotheses	Path coefficient	*p* values	*f* ^2^	Hypotheses supported
H1a: Perceived severity → Identity	0.118	0.000	0.014	Yes
H1b: Perceived benefits → Identity	0.164	0.000	0.027	Yes
H1c: Perceived barriers → Identity	0.049	0.162	0.002	No
H1d Cue to action → Identity	0.137	0.000	0.019	Yes
H2: Identity → Socially responsible consumption	0.571	0.000	0.474	Yes
H3a: Perceived severity → Socially responsible consumption	0.027	0.349	0.001	No
H3b: Perceived benefits → Socially responsible consumption	0.028	0.342	0.001	No
H3c: Perceived barriers → Socially responsible consumption	0.085	0.006	0.011	Yes
H3d: Cue to action → Socially responsible consumption	0.049	0.080	0.004	No

With the path coefficients, the tests of hypotheses 1a to 3d of the study were carried out. First, the effects of the stimuli on the organism (social identity) were evaluated, for which hypothesis tests 1a, 1b, 1c, and 1d were performed. Hypotheses 1a, 1b, and 1d are tested. Perceived severity significantly influences social identity (*β* = 0.118, *p* = 0.000). In turn, perceived benefits significantly influence social identity (*β* = 0.164, *p* = 0.000). Furthermore, the cue to action significantly influences social identity (*β* = 0.137, *p* = 0.000). Although hypothesis 1c is not tested, perceived barriers do not significantly influence social identity (*β* = 0.049, *p* = 0.162).

Second, the effect of the organism (social identity) on the response (socially responsible consumption) was evaluated using hypothesis 2. Finally, Hypothesis 2, which postulates that social identity significantly influences socially responsible consumption, is tested (*β* = 0.571, *p* = 0.000).

Third, the effects of the stimuli on the response (socially responsible consumption) were evaluated through hypotheses 3a, 3b, 3c, and 3d. In the case of hypotheses 3a, 3b, and 3d, there is no significant influence from perceived severity (*β* = 0.027, *p* = 0.349), perceived benefits (*β* = 0.028, *p* = 0.342), and cue to action (*β* = 0.049, *p* = 0.080) in socially responsible consumption, so these hypotheses are not proven. On the other hand, while hypothesis 3c is tested, although perceived barriers do not significantly influence social identity, they significantly influence socially responsible consumption (*β* = 0.085, *p* = 0.006).

### 4.3. Mediation analysis

For hypothesis tests, 4a, 4b, 4c, and 4d, the mediation effect of social identity (organism) between the relationship of external stimuli and socially responsible consumption (response) was evaluated, see Table 8. Two steps were proposed by Nitzl et al. (2016) with a Bootstrapping analysis to assess effect of PLS-SEM mediation. The first step consisted in determining the significance of the indirect effect and the second step was determining the type of mediation by evaluating the significance of the direct effect. If the indirect and direct effects are significant, there is a partial mediation, and when only the indirect effect is significant, it is a total mediation (Zhao et al., 2010). Therefore, only hypotheses 4a, 4b, and 4d are tested. Social identity significantly mediates the relationship between perceived severity and socially responsible consumption (*β* = 0.067, *p* = 0.000). Likewise, social identity significantly mediates the relationship between perceived benefits and socially responsible consumption (*β* = 0.094, *p* = 0.000). Moreover, social identity significantly mediates the relationship between cue to action and socially responsible consumption (*β* = 0.078, *p* = 0.000). However, hypothesis 4c is not tested, social identity does not mediate the relationship between perceived barriers and socially responsible consumption (*β* = 0.028, *p* = 0.166).

**Table 8:** Mediation analysis results.

Hypotheses	Indirect effect	*t* statistics	*p* values	Hypotheses supported	Type of mediation
H4a: Perceived severity → Identity → Socially responsible consumption	0.067	3.506	0.000	Yes	Indirect only (Full mediation)
H4b: Perceived benefits → Identity → Socially responsible consumption	0.094	4.842	0.000	Yes	Indirect only (Full mediation)
H4c: Perceived barriers → Identity → Socially responsible consumption	0.028	1.384	0.166	No	No mediation
H4d: Cue to action → Identity → Socially responsible consumption	0.078	4.119	0.000	Yes	Indirect only (Full mediation)

The model’s fit was evaluated using the standardized mean square residual (SRMR) values considering values less than 0.08 to confirm a good fit (Benitez et al., 2020). The SRMR value of the model was 0.050, which was less than 0.08, so it is considered a good model fit.

### 4.4. Multigroup analysis

To test hypothesis 5, it was necessary to carry out a multigroup analysis; however, before said analysis, the calculation of the measurement invariance of composite models (MICOM) was carried out to corroborate that the categorical variable causes the changes in the structural model, in this case, the age; this analysis tested the measurement invariances between groups (Hair et al., 2021). The MICOM calculation was integrated into three stages, the confirmation of the configural invariance, the compositional invariance, and the equality of means and variances of the composites (Henseler et al., 2016). First, configural invariance was confirmed since the same indicators and scales were used in the four age groups, the same data treatment, and the same algorithms (Hair et al., 2021). Second, for the confirmation of compositional invariance, a permutation analysis was carried out with 1,000 permutations, and it was confirmed *p*-value of the correlations of the constructs was greater than 0.05 to guarantee that there are no differences between the composites and thus prove the invariance of the composites (Hair et al., 2021). Table 9 shows the composite invariance of the constructs between the groups that had significant differences between their path coefficients. Third, since all the composites in the groups with significant differences had compositional invariance, equality of means and variances between the composites in the groups was confirmed. It was examined that the value of the difference between means and variances was greater than 0.05 to prove the equality of means and variances and with it full measurement invariance (Henseler et al., 2016). However, in some cases, equality of means and variances was not confirmed, so in some cases, there was only partial measurement invariance; this happens when only the second step is completed. If there is at least partial measurement invariance, it is possible to perform a multigroup analysis (Hair et al., 2021).

**Table 9:** MICOM results.

		Generation z-Generation y						Generation z-Generation x						Generation y – Generation x					
Measurement invariance		SEP	BEP	BAP	CA	IDE	CSR	SEP	BEP	BAP	CA	IDE	CSR	SEP	BEP	BAP	CA	IDE	CSR
Configural invariance		Yes	Yes	Yes	Yes	Yes	Yes	Yes	Yes	Yes	Yes	Yes	Yes	Yes	Yes	Yes	Yes	Yes	Yes
Compositional invariance	*c* value	0.975	0.996	0.977	1.000	1.000	1.000	0.977	0.998	1.000	1.000	1.000	1.000	0.926	0.994	0.987	1.000	1.000	1.000
	*p-*Value	0.252	0.408	0.352	0.502	0.643	0.917	0.196	0.571	0.881	0.483	0.595	0.528	0.077	0.295	0.403	0.240	0.605	0.498
	Compositional invariance	Yes	Yes	Yes	Yes	Yes	Yes	Yes	Yes	Yes	Yes	Yes	Yes	Yes	Yes	Yes	Yes	Yes	Yes
Equality of means and variances	Means difference	−0.227	0.214	0.035	−0.171	−0.105	−0.118	−0.208	0.144	0.075	−0.252	−0.130	0.039	0.018	−0.079	0.043	−0.084	−0.025	0.157
	*p-*Value	0.023	0.04	0.732	0.092	0.3	0.226	0.019	0.107	0.421	0.005	0.153	0.637	0.887	0.495	0.720	0.465	0.829	0.175
	Equality of means	No	No	Yes	Yes	Yes	Yes	No	Yes	Yes	No	Yes	Yes	Yes	Yes	Yes	Yes	Yes	Yes
	Variance difference	0.063	0.257	−0.157	0.064	−0.025	0.139	0.086	0.003	−0.154	−0.091	−0.135	−0.042	0.066	−0.260	−0.008	−0.154	−0.113	−0.182
	*p-*Value	0.618	0.027	0.184	0.585	0.874	0.405	0.400	0.983	0.172	0.352	0.335	0.767	0.625	0.097	0.951	0.166	0.567	0.345
	Equality of variances	Yes	No	Yes	Yes	Yes	Yes	Yes	Yes	Yes	Yes	Yes	Yes	Yes	Yes	Yes	Yes	Yes	Yes
Type of measurement invariance		Partial measurement invariance	Partial measurement invariance	Full measurement invariance	Full measurement invariance	Full measurement invariance	Full measurement invariance	Partial measurement invariance	Full measurement invariance	Full measurement invariance	Partial measurement invariance	Full measurement invariance	Full measurement invariance	Full measurement invariance	Full measurement invariance	Full measurement invariance	Full measurement invariance	Full measurement invariance	Full measurement invariance

SEP, perceived severity; BEP, perceived benefits; BAP, perceived barriers; CA, cue to action; IDE, identity; CSR, socially responsible consumption.

A bootstrapping analysis was performed to determine the path coefficients of the relationships of the research model between groups and their significance (Hair et al., 2017, 2021). Table 10 shows these results. Differences in path coefficients between groups were analyzed by multigroup analysis (MGA; Henseler et al., 2009). The results of the multigroup analysis are shown in Table 11. There is a significant difference in the cue to action and social identity relationship in age groups 1 and 2, which correspond to generation Z and generation Y. A significant difference was also found in groups 1 (generation Z) and 3 (generation X) in the relationships between cue to action and social identity and between perceived benefits and socially responsible consumption. Moreover, a significant difference was found in groups 2 (generation Y) and 3 (generation X) in the relationship between cue to action and social identity. Therefore, hypothesis 5 is partially tested because a difference was only found in four relationships between the generational groups.

**Table 10:** Results of the structural model of the groups.

Relationships	Age 1		Age 2		Age 3		Age 4	
	Path coefficient	*p*-Value	Path coefficient	*p*-Value	Path coefficient	*p*-Value	Path coefficient	*p*-Value
Cue to action → Identity	0.121	0.004	−0.126	0.163	0.280	0.000	−0.006	0.978
Cue to action → Socially responsible consumption	0.059	0.076	−0.013	0.883	0.054	0.416	0.045	0.838
Identity → Socially responsible consumption	0.622	0.000	0.418	0.000	0.567	0.000	0.357	0.328
Perceived barriers → Identity	−0.003	0.942	0.139	0.161	0.119	0.110	0.317	0.195
Perceived barriers → Socially responsible consumption	0.129	0.001	0.054	0.647	0.046	0.519	−0.118	0.698
Perceived benefits → Identity	0.217	0.000	0.187	0.043	0.068	0.381	0.411	0.125
Perceived benefits → Socially responsible consumption	−0.021	0.565	0.047	0.608	0.126	0.040	0.096	0.709
Perceived severity → Identity	0.116	0.004	0.166	0.045	0.097	0.178	0.423	0.122
Perceived severity → Socially responsible consumption	0.010	0.790	0.107	0.299	0.030	0.650	0.158	0.669

**Table 11:** Multigroup analysis (MGA).

Relationship	Difference (generation Z– generation Y)	Difference (generation Z – generation X)	Difference (generation Z– baby boomers)	Difference (generation Y-generation X)	Difference (generation Y – baby boomers)	Difference (generation X-baby boomers)	Generation Z – generation Y *p*-value	Generation Z – generation X *p*-value	Generation Z – baby boomers’ *p*-value	Generation Y-generation X *p*-value	Generation Y– baby boomers’ *p*-value	Generation X-baby boomers’ *p*-value
Cue to action → Identity	0.247	−0.159	0.127	−0.406	−0.120	0.286	0.013	0.047	0.574	0.001	0.613	0.207
Cue to action → Socially responsible consumption	0.072	0.005	0.014	−0.067	−0.058	0.009	0.451	0.952	0.999	0.548	0.767	0.985
Identity → Socially responsible consumption	0.204	0.055	0.265	−0.149	0.061	0.210	0.057	0.549	0.466	0.261	0.907	0.586
Perceived barriers → Identity	−0.143	−0.122	−0.321	0.021	−0.178	−0.198	0.185	0.162	0.222	0.818	0.460	0.409
Perceived barriers → Socially responsible consumption	0.074	0.082	0.246	0.008	0.172	0.164	0.552	0.308	0.448	0.903	0.623	0.638
Perceived benefits → Identity	0.030	0.149	−0.195	0.119	−0.225	−0.344	0.781	0.078	0.308	0.289	0.295	0.194
Perceived benefits → Socially responsible consumption	−0.068	−0.147	−0.117	−0.079	−0.049	0.030	0.495	0.042	0.569	0.480	0.769	0.995
Perceived severity → Identity	−0.049	0.020	−0.306	0.069	−0.257	−0.326	0.559	0.819	0.245	0.506	0.301	0.234
Perceived severity → Socially responsible consumption	−0.097	−0.021	−0.149	0.077	−0.051	−0.128	0.346	0.767	0.639	0.505	0.774	0.664

### 4.5. Alternative conceptual model analysis

**Figure 3:**
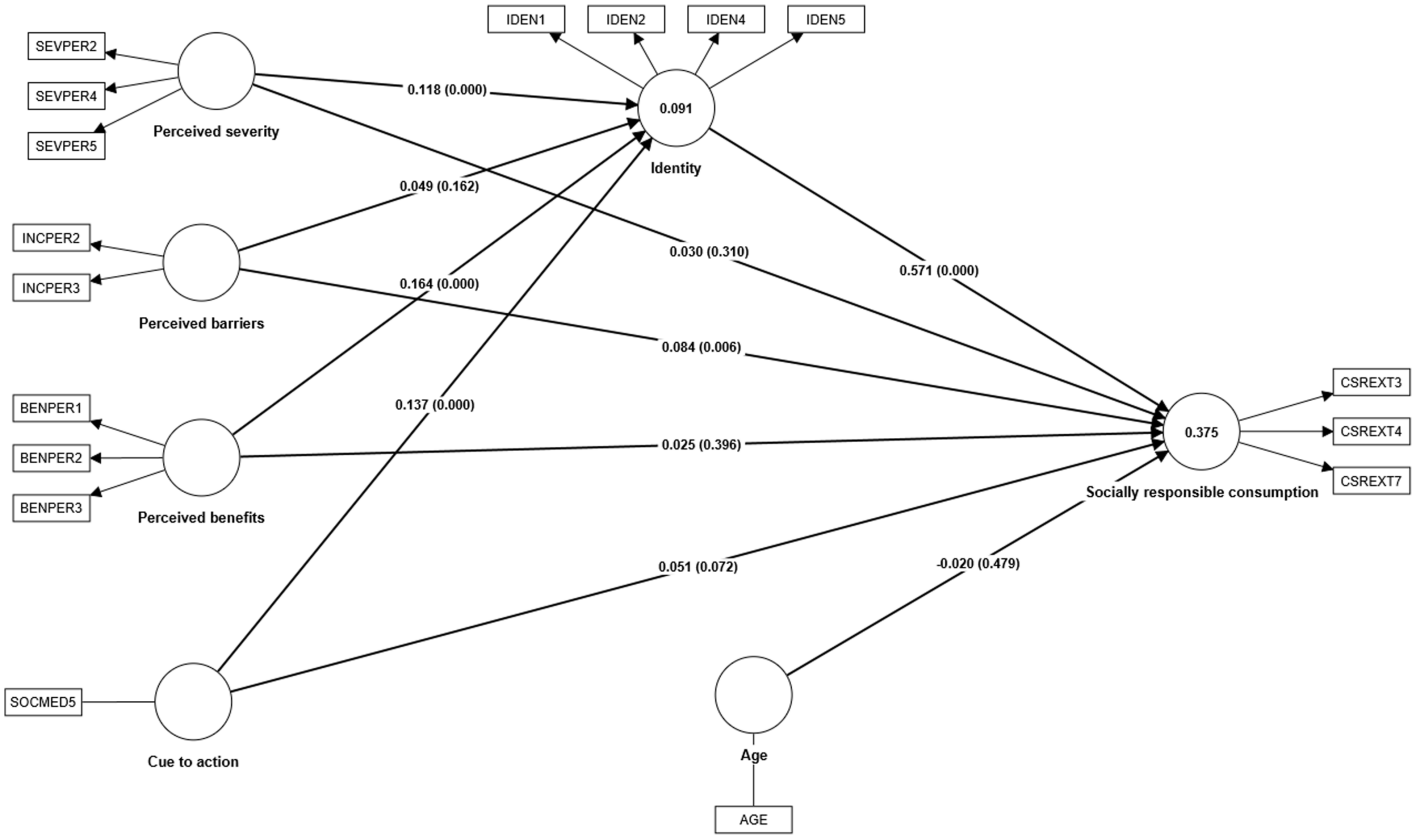
Alternative conceptual model.

An alternative conceptual model with an additional exogenous variable was analyzed as Stewart et al. (2010) did in constructing an alternative conceptual model. Like these authors, age was included as an exogenous variable of the model since there were differences in some relationships due to generational change. However, age did not significantly influence on socially responsible consumption and its effect was negative (*β* = −0.020, *p* = 0.479) as shown in Table 12. Furthermore, this variable did not affect the degree of explanation of the endogenous variable since the determination coefficient remained the same as in original model (*R*^2^ = 0.375) as seen in Figures 2, 3.

**Table 12:** Results of the alternative conceptual model.

Relationship	Path coefficient	*p* values	*f* ^2^
Age → Socially responsible consumption	−0.020	0.479	0.001
Perceived severity → Identity	0.118	0.000	0.014
Perceived benefits → Identity	0.164	0.000	0.027
Perceived barriers → Identity	0.049	0.162	0.002
Cue to action → Identity	0.137	0.000	0.019
Identity → Socially responsible consumption	0.571	0.000	0.475
Perceived severity → Socially responsible consumption	0.030	0.310	0.001
Perceived benefits → Socially responsible consumption	0.025	0.396	0.001
Perceived barriers → Socially responsible consumption	0.084	0.006	0.010
Cue to action → Socially responsible consumption	0.051	0.072	0.004

## 5. Discussion

According to the results of this investigation, it was shown that most of the external stimuli caused by COVID-19 had a significant effect on the organism (social identity), which in turn, leads to a response (socially responsible consumption of food). The perceived severity that the consumer has, their perception of fragility in the face of COVID-19, as well as the perceived benefits that socially responsible foods have on the vulnerability condition that COVID-19 causes in them, in addition to participating in social networks related to the subject triggers the consumer to improve their self-perception as socially responsible and consequently make more significant efforts to buy food from companies that promote ecological practices and local commerce. This supports what is postulated in the stimulus organism response model (Mehrabian and Russell, 1974), which indicates that the external stimulus affects the internal state of the organism and therefore it has a response.

Previous studies also explain purchasing behavior during the health contingency through this theoretical model (Liu et al., 2021; Yin et al., 2021), which also provides more significant support for the applicability of the SOR model in consumption. Although in contrast to them, this research combines the SOR model with the health belief model that explains purchasing behavior not only during the pandemic but also extends the understanding to define types of stimuli caused by a condition that harms the health of society.

Unlike studies previously carried out during the COVID-19 pandemic that analyze stimuli considered in this research separately, this study made it possible to integrate all of them into a model that could explain purchasing behavior through the postulates of the health belief model. Laato et al. (2020) in their research on purchasing behavior, suggested that the exposure to online information is the environmental stimulus caused at the beginning of the pandemic that may be similar to the cue to action evaluated in this study. However, they indicate that this stimulus external stimulus causes an effect on the perceived severity that, in this study, was proposed as an external stimulus. In turn, Wang et al. (2021), through the SOR model to explain the intention to purchase organic food, only considered an external stimulus, the perceived severity of those tested in this study that affects the organism.

Other studies, which analyze different behavior under the SOR model, have found that some of the stimuli proposed in this research also affect the internal state of the consumer measured as a psychological process, such as that of Liu et al. (2020). Who studied the effect of the information generated on social networks on the person, although like consumer studies, they did not jointly analyze various stimuli that COVID-19 can cause and that this research evaluated. So, through the SOR model and the health belief model, it is possible to analyze the behavior of consumers in similar pandemic situations.

Therefore, with the results found in this study, it is possible to affirm that the health belief model is an appropriate framework to evaluate the effect of COVID-19 as an external stimulus that affects the individual’s internal state. Specifically, perceived severity, benefits, and cue to action increased consumers’ perception of socially responsible individuals. If they felt fragile in the face of the COVID-19 disease, they noticed changes in their health and eating habits due to COVID-19. They perceived that a diet based on organic, agroecological, and fair consumption foods reduced the probability of contagion and complications of the disease. In addition, they participated in social network groups where people sold or consumed organic food, and perceived that they were, acted, and saw them as socially responsible. Therefore, the opportunity has been opened to rethink how to address these problems to reorient them and aim at building a more sustainable future, integrating sustainable food and agriculture into development strategies (FAO et al., 2021).

The social identity of the socially responsible consumer causes them to put more effort into purchasing socially responsible food. Similar results were found by Talwar et al. (2021) since they proved that when the consumer perceives himself as ethical, he will purchase organic food. However, the stimuli that trigger their behavior are others, health consciousness, and food safety concerns. In contrast, in this study, the stimuli caused by the pandemic were considered from the health belief model. With this model, it is possible to explain behavior when there is a context of uncertainty and risk to the health of a population.

According to other contributions to the literature, it has been seen that COVID-19 caused changes in the decision to purchase food that varied according to age or gender, which were also related to their emotional state since confinement brought psychological consequences in consumers such as tension, fatigue, depression, anxiety to mention a few (Di Renzo et al., 2020; Russo et al., 2021). What is related to what was postulated by the SOR, which, in turn, was evidenced in this study because the external stimuli caused by COVID-19 affect the internal state of the consumer, in this case, the social identity, and therefore changes purchasing behavior, which was found to increase.

A positive effect of social identity on purchasing behavior was found. However, only perceived barriers were found to have a positive and significant effect on behavior, although their effect size was null. Although consumers need more time to obtain socially responsible foods, and it is sometimes difficult to distinguish them from conventional foods, they continue to buy these products because the other stimuli caused by COVID-19 lead people to increase their purchases. As other authors confirm, the force of the COVID event is large enough so that, through the changes caused in the body, the purchase of food with health and environmental benefits is generated (Yin et al., 2021).

The results also prove that the SOR has served as a framework to test the mediating role of the organism, which is consistent with the findings of Yu et al. (2021) and Liu and Zheng (2019) on the purchase of organic food, but which did not analyze the effect of COVID-19 as the external stimulus. The former examined the mediating effect of trust on the relationship between image and purchase intention, and the latter examined the mediating effect of cognition on the relationship between food safety incidents, environment orientation, and health orientation with organic foods purchase intention. According to the findings and in addition to the above, through the SOR, the mediating role of the organism between the stimuli and the purchasing behavior can be evaluated.

Depending on the age of consumers, COVID-19 has caused different changes in their food consumption habits among young people with an impulsive approach and older people with a conservative approach (Russo et al., 2021). In addition, for millennials, social identity is a factor that explains why the consumer has socially responsible purchasing behavior, such as buying from organizations that respect the environment, have ethical practices, and strive for socially responsible causes (Johnson and Chattaraman, 2021). It has been indicated that the type of social networks used in purchases depends on age; those of generation X prefer networks for professional use or those that have been in use for more time, such as Linkedin or Skype, while those of Generation Z prefer more recently created networks such as Instagram or Tik Tok (Taha et al., 2021).

In general, differences between generations can be seen in this research that is consistent with these studies; however, specifically, the changes were found in the effects of social networks on social identity. The impact of social networks was more significant among consumers of generation Z than those of generation Y; in turn, this impact was more significant in generation X than generation Z and Y, which partly contrasts with the previous literature. Younger consumers are expected to be more affected by social networks due to their familiarity with digital media; young people of generation Y were the first that digitalization affected their lives and work (Bolton et al., 2013).

However, the findings revealed that for Gen Xers, networks significantly affect how they see themselves as socially responsible consumers. Previous studies, such as the one by Severo et al. (2019), found that the effect of social networks on environmental awareness is less in adults of generation Y than in those of generation X. However, the effect of social networks on their social responsibility awareness is more remarkable. This way can help explain the findings since, in this research the social and environmental aspects of the concept of social identity were not distinguished, this variable contemplates both. The ecological element may be more critical for generation X than for the generations. Still, it would have to be proven in future research that distinguishes the effect of social networks in each of these dimensions of the social identity variable.

## 6. Conclusion

The results confirm that the stimulus-organism response and health belief models are appropriate for analyzing socially responsible consumption. Furthermore, since most of the external stimuli (caused by COVID-19) analyzed in this research from the health belief model. They had a positive and significant effect on the organism, measured as the social identity of the consumer, and this, in turn, led to socially responsible consumption as postulated by the stimulus organism response model.

Perceived severity, perceived benefits, and cue to action positively affect consumer social identity. For example, when the consumer perceives that COVID-19 affects his health, changes his eating habits, and makes him fragile, the importance he places on being socially responsible will increase. In addition, if the individual considers the benefits, he will have by consuming socially responsibly, his social identity as a consumer will be strengthened. Moreover, if he is a member of groups in social networks related to socially responsible consumption, his thoughts about social responsibility will be more significant, as well as his self-perception as socially responsible.

This research proves that social identity has a large effect on socially responsible consumption and a positive and significant effect on it. Therefore, if individuals consider that they are socially responsible and that this is important to them, make an effort to support and buy from food companies that have green practices such as waste management and recycling and that also promote local commerce.

Likewise, through social identity, external stimuli positively affect socially responsible food consumption. The perceived severity, the perceived benefits, and the cue to action positively influence the existence of socially responsible consumption only when the social identity of the individual as socially responsible is involved; if this variable is not present, external stimuli have no effect on consumption socially responsible.

The results of this research have implications for the design of public policies or marketing strategies that can encourage socially responsible consumption according to the age of the individuals since it was found that age affects how consumers are perceived as socially responsible. Because they participated in social networks, future campaigns aimed at adults of generation Z and X, whose objective is to promote socially responsible consumption, could consider social network groups as the main source of communication. The results prove that the effect of social networks on social identity is more significant among young adults of Generation Z than generation Y and that this effect is more significant among adults of generation X than generation Z and Y. In this way, their social identity as socially responsible consumers could be increased, and consequently, their socially responsible food consumption would increase. On the other hand, decision-makers and public policymakers have to consider that the COVID-19 pandemic exposes the fragility of food security and nutrition progress.

In Mexico, various groups and organizations develop actions and projects to strengthen sustainable food consumption. So, there are many solutions that unites food process transformation initiatives. It is necessary to identify the contribution of each of the actors involved in the process of healthy eating. Educators, food producers, consumers, society, and food marketers are among them. Sustainable food consumption is based on food education, whose purpose is to develop healthy eating habits, which is achieved by properly focusing education adequately that promote the consumption of local and seasonal foods, establish urban gardens, and promote creativity in the preparation of local foods in healthy dishes-shortening the value chains and establishing marketing channels without intermediaries between consumers and producers, where producers and marketers have an essential role.

## 7. Limitations and future research

One of the study’s main limitations was the sample size of the last generational group (baby boomers) since it was well below the size of the other groups; possibly, for this reason, no significant differences were found with this group. That was because the data collection was voluntary and random without including any sample segmentation criteria to avoid a disproportionate representation of socially responsible consumers. That led to greater participation by young consumers than by older adults. Therefore, it is recommended that future research analyze whether this generational group with a larger sample size has significant differences with younger groups in their socially responsible food consumption, given that differences were found between the younger groups.

Another limitation of the research was the analysis of the cue to action variable with a single item that measured individuals’ perceptions about their participation in social network groups related to the theme. Because perception was evaluated, it is possible to obtain a bias in the results due to socially desirable responses, which is expected to find when analyzing ethical behaviors. For this reason, it is recommended that future research use numerical values as frequencies of participation in groups related to the theme for the measurement of the variable or other types of approaches, such as experiments, to corroborate the results of this research.

Another limitation of the study was the place of data collection; an urban area that was the metropolitan area of Mexico City was considered for the study. Future research may include a comparison between rural and urban areas to explore whether there is any difference in consumers’ perception due to the degree of urbanization in the locality. In addition to this, another limitation of the study was the period, this research is cross-sectional, so it is suggested that future research carry out a longitudinal study to test the model over time and analyze its effectiveness.

According to the findings, future research may analyze the effect of social networks on the social and environmental aspects of social identity across generations since, in this way, generation-specific marketing strategies could be generated. Additionally, the type of social network is recommended to evaluate the differences since previous studies have shown its relevance when analyzing purchasing behavior. However, its effect on socially responsible food purchases has yet to be seen.

## Data availability statement

The original contributions presented in the study are publicly available. This data can be found here: [https://data.mendeley.com/datasets/wksx357hch/1].

## Ethics statement

The studies involving human participants were reviewed and approved by Universidad Panamericana, Facultad de Ciencias Económicas y Empresariales. The patients/participants provided their written informed consent to participate in this study.

## Author contributions

SNL-H: methodology, interpreted results, and discussion. AT-B: introduction. SNL-H and AT-B: conceptualization, literature review, conclusions, and editing. AT-B and AM-V: data collection and review. All authors listed have made a substantial, direct, and intellectual contribution to the work, and approved it for publication.

## Conflict of interest

The authors declare that the research was conducted in the absence of any commercial or any financial relationships that could be construed as a potential conflict of interest.

## Publisher’s note

All claims expressed in this article are solely those of the authors and do not necessarily represent those of their affiliated organizations, or those of the publisher, the editors and the reviewers. Any product that may be evaluated in this article, or claim that may be made by its manufacturer, is not guaranteed or endorsed by the publisher.
